# Lessons from Ebola: Sources of Outbreak Information and the Associated Impact on UC Irvine and Ohio University College Students

**DOI:** 10.1371/currents.outbreaks.f1f5c05c37a5ff8954f38646cfffc6a2

**Published:** 2016-08-25

**Authors:** Thrissia Koralek, Miryha G. Runnerstrom, Brandon J. Brown, Chukwuemeka Uchegbu, Tania B. Basta

**Affiliations:** Program in Public Health, University of California Irvine, Irvine, California, USA; Program in Public Health, University of California Irvine, Irvine, California, USA; Center for Healthy Communities, Department of Social Medicine and Population Health, University of California Riverside School of Medicine, Riverside, California, USA; Department of Social and Public Health, College of Health Sciences and Professions, Ohio University, Athens, Ohio, USA; Department of Social and Public Health, College of Health Sciences and Professions, Ohio University, Athens, Ohio, USA

## Abstract

**Objectives.** We examined the role of outbreak information sources through four domains: knowledge, attitudes, beliefs, and stigma related to the 2014 Ebola virus disease (EVD) outbreak.

**Methods.** We conducted an online survey of 797 undergraduates at the University of California, Irvine (UCI) and Ohio University (OU) during the peak of the outbreak. We calculated individual scores for domains and analyzed associations to demographic variables and news sources.

**Results.** Knowledge of EVD was low and misinformation was prevalent. News media (34%) and social media (19%) were the most used sources of EVD information while official government websites (OGW) were among the least used (11%). Students who acquired information through OGW had higher knowledge, more positive attitudes towards those infected, a higher belief in the government, and were less likely to stigmatize Ebola victims.

**Conclusions.** Information sources are likely to influence students’ knowledge, attitudes, beliefs, and stigma relating to EVD. This study contains crucial insight for those tasked with risk communication to college students. Emphasis should be given to developing effective strategies to achieve a comprehensive knowledge of EVD and future public health threats.

## **** ****INTRODUCTION

Given the ongoing and unpredictable threat to human health posed by recent emerging infectious diseases (EID), the appearance and exportation of future novel pathogens is expected to continue and, with it, our dependence on reliable risk communication to assist in disease containment. Rapid pathogenic genetic changes and travel-associated infections have contributed to the difficulty containing the spread of diseases.^1^ Apart from the ongoing Zika virus epidemic, the most salient example of EID in our closely interconnected world is the unprecedented 2014 Ebola virus disease (EVD) epidemic in West Africa.

Although the eradication of Smallpox and Rinderpest virus remains a momentous achievement in public health history,^2^ the recent EVD outbreak provided a reminder that pathogens are still capable of crossing national boundaries and posing a global threat.^3^ Over 2 years have passed since the World Health Organization (WHO) publicly announced the EVD outbreak in West Africa, on March 23rd, 2014.^4^ Since the beginning of the outbreak, the total number of reported cases have reached over 28,646 and caused more than 11,323 deaths within six different countries: Guinea, Liberia, Sierra Leone, Mali, Nigeria, and the United States (U.S.).^5^

There is evidence that demonstrates American college students’ lack of disease knowledge during the height of an epidemic. During the 2009 H1N1 influenza pandemic, it was estimated that between August 2009 and April 2010, over 95,500 cases of influenza-like illnesses were reported at 170 American college institutions.^6^ Although a defining characteristic of this pandemic was the disproportionate prevalence among those younger than 25 years old,^7^^,^ 8 researchers have found that college students showed insufficient knowledge and remained ignorant of the facts surrounding the H1N1 vaccine and the effectiveness of flu-prevention behaviors.^9^ A gap in H1N1 influenza knowledge and misconceptions regarding symptoms, treatments, and modes of transmission were widespread among this population.^10^^,^ 11 Unfortunately, lack of disease characteristics’ knowledge facilitates preventable disease transmission, health behavior inaction,^12^ and fear of disease, which can lead to social stigmatization.^13^

The likelihood of international students introducing novel diseases to American college campuses cannot be ignored. During the 2013-2014 academic year, over 886,000 international students attended American post-secondary institutions.^14^ As enrollment in American colleges and universities continues to grow, with roughly 20.2 million students expected to attend post-secondary institutions during fall 2015,^15^ so does the risk for the emergence of novel diseases on campuses. Furthermore, American college students engage in international travel as a way of fostering professional growth and cross-cultural experiences. During the 2012-2013 academic year, more than 289,000 students participated in study abroad programs.^16^ Due to the international mobility of students, there is a heightened vulnerability for students to be exposed to infectious diseases worldwide and as a consequence, to introduce and allow for college campuses to act as disease outbreak centers.

Accurate knowledge of disease symptoms and modes of transmission can serve as crucial information during an outbreak. Although recent studies in Nigeria, Sudan, and the U.S. have highlighted Ebola-related gaps in knowledge and misconceptions,^17^^,^ 18 ^,^ 19 ^,^ 20 limited information about American college students knowledge of the 2014 EVD outbreak exists. Similarly, college students’ news source preferences during an outbreak may have an effect on their knowledge of the disease, but this correlation hasn’t been adequately explored.

To address this gap, we examined various risk communication sources utilized by college students during the 2014 EVD outbreak and their impact on students’ knowledge, attitudes, beliefs, and stigma towards Ebola victims.

## METHODS

**Participants**

A convenience sample was recruited from two American Universities of far geographical proximity, University of California Irvine (UCI) and Ohio University (OU). Eligibility criteria included being at least 18 years old and a current undergraduate student. Eligible students provided informed consent prior to completion of the survey.

**Procedure**

The sample was recruited through mass department e-mails and through university-specific social networking sites such as UCI and OU Facebook club pages. Data collected were examined and compared in order to assess news sources impact on students’ knowledge, attitudes, beliefs, and stigma related to EVD. Demographic variables including gender, age, academic major, and level of education were some of the key factors that were considered while comparing survey data from both universities. For purposes of analysis, academic majors were categorized into “biological sciences college exposure” and “non-biological sciences college exposure.” The primary criterion for majors to be included into the “biological sciences college exposure” category was having exposure to any biological science course as part of students' academic major requirements. This would account for biases arising from previous Ebola knowledge acquired through these courses. All others were categorized as “non-biological sciences college exposure”.

**Data Collection and Measures**

The survey administered between February and April 2015 at both universities consisted of 53 items across a total of six sections: news source preferences, Ebola knowledge, stigma towards Ebola victims, beliefs regarding the U.S. Government’s association with the 2014 outbreak, attitudes towards EVD, and demographic variables.

*Ebola knowledge.* A total Ebola knowledge score was computed by summing correct responses for 24 statements regarding Ebola facts, symptoms, and modes of transmission. Answer options for each statement were “True,” “False,” and “Not sure.” Responses were scored as correct or incorrect according to EVD information released through the CDC website.^21^ Responses were summarized generating a maximum score possible of 24 (0/incorrect vs 1/correct). Higher scores indicated a better knowledge of EVD.

*Attitudes.* Responses were analyzed as reflecting positive or negative attitudes towards EVD. There was a total of 9 statements that examined respondents’ attitudes. Participants were asked to select on a 4-point Likert scale ranging from strongly disagree=1 to strongly agree=4. Responses that revealed a general awareness of the disease and the severity of the outbreak in the U.S. were considered positive attitude responses towards EVD, each coded as 1 (e.g., somewhat or strongly agree on responses to “Flu is a bigger threat than Ebola in the U.S.”), all other responses were coded as zero. Higher means suggested a more positive attitude towards EVD.

*Beliefs.* We assessed participants’ beliefs concerning the U.S. Government’s involvement with the 2014 Ebola epidemic and the government’s ability to fight the outbreak with 3 items: “Ebola is a government conspiracy created to get rid of a particular race,” “There is a cure for Ebola but the government is keeping it from the public,” and “The CDC is doing all it can to control the epidemic and stop the spread of Ebola in the U.S.” Answers varied from “True,” “False,” and “Not sure.” Answers options of False, False, and True to above items were coded as 1, respectively. These answers options represent a “positive belief”, all other responses were scored as zero, representing a “negative belief.” Responses were summarized, resulting in a belief score ranging from 0 to 3. Higher scores indicated a higher level of trust in the government system.

*Stigma.* We asked respondents how much they agreed or disagreed with seven statements that analyzed stigma towards Ebola victims. Response options were coded as 1= “Strongly Disagree,” 2= “Somewhat Disagree,” 3= “Somewhat Agree,” 4= “Strongly Agree.” We used means to summarize responses. Higher means indicated higher levels of stigma towards Ebola victims. Moreover, to examine associations between respondents’ stigma and the effect of stigma on participant knowledge of Ebola, we created a dichotomous variable for each statement with 1 for “Somewhat Agree” and “Strongly Agree”, and zero for “Strongly Disagree” and “Somewhat Disagree”.

*Statistical* *analyses.* The data were analyzed by using Stata version 13.1 (StataCorp LLC, College Station, TX). No differences in characteristics of both subgroups were found; therefore we merged data sets from UCI and OU to examine Ebola knowledge, attitudes, beliefs, and stigma as well as its associations with news sources and demographic variables. We calculated arithmetic means, frequencies, percentage values, and standard deviations by using descriptive statistics. We used one-way analysis of variance (ANOVA) to examine various mean differences between groups.

## RESULTS

A total of 797 undergraduates were included in the study, 514 students from UCI and 283 students from OU. Of these, 546 (72%) were female, 240 (32%) were seniors in college, 557 (74%) belonged to “non-biological sciences college exposure” academic majors, and 331 (42%) were 20 to 21 years of age. Students in the study averaged 20.9 years of age (SD=4.0; range=18-57). Those in the “non-biological sciences college exposure” major category represented a total of 30 academic programs.

At the time that the survey was conducted, the undergraduate population size from UCI and OU was 23,807 35 and 17,965,^36^ respectively. According to the criteria used for analyzing academic majors, 21% of UCI majors would be considered as a “biological sciences college exposure” academic major, while 6.4% of OU academic majors. Within our sample, 109 (23% of UCI sample) and 83 (29% of OU sample) respondents were representative of “biological sciences college exposure” academic majors. Overall, 192 (26%) respondents belonged to “biological sciences college exposure” academic majors which included undergraduates in public health, biological sciences, biochemistry, pharmaceutical sciences, and nursing.

**Risk Perception**

Data obtained from both universities indicate that the majority of participants (81%) perceived a low personal risk of acquiring EVD. When asked to rank how afraid they were of contracting EVD using a Likert Scale ranging from 1 (not at all afraid) to 10 (very afraid), 81% of responses ranged from 1 to 5 (not at all afraid to moderately afraid), with 37% of participants choosing category 1. Female respondents perceived themselves as being at higher risk in comparison to males (Mean=3.25 vs. 2.63 respectively; P= .003).

**Ebola Knowledge**

Our findings suggest that although nearly all respondents (96%) had heard of EVD, gaps in Ebola knowledge and misinformation regarding transmission methods were prevalent among this sample. Across all participants, Ebola knowledge was generally low; the mean percentage correct score on the Ebola knowledge section was 49%; the mode knowledge score was 15 on a 24-point scale (n=81). Additionally, 34 respondents (4%) reported not having heard of EVD prior to the survey.

Based on a maximum score of 24, the mean Ebola knowledge scores obtained for UCI and OU students was 10.9 (SD=5.3) and 13.5 (SD=4.4), respectively. Among both student populations, 25% were aware that Ebola can remain active in the semen fo three months after infection. Additionally, 34% were aware that the Ebola virus couldn’t be transmitted through mosquitoes and 31% understood that asymptomatic carriers on an airplane could not transmit EVD.

The majority of all respondents (71%) correctly recognized that EVD is characterized by having an incubation period of up to 21 days. Findings also indicate that students were aware that the Ebola virus could be transmitted by direct contact with the body fluids of an infected person, with 91% of students answering this question correctly. While the majority of participants were able to identify “fever” as an EVD symptom (88%), a lower percentage correctly identified “bleeding” as another symptom (60%) (Table1).

**Figure:**
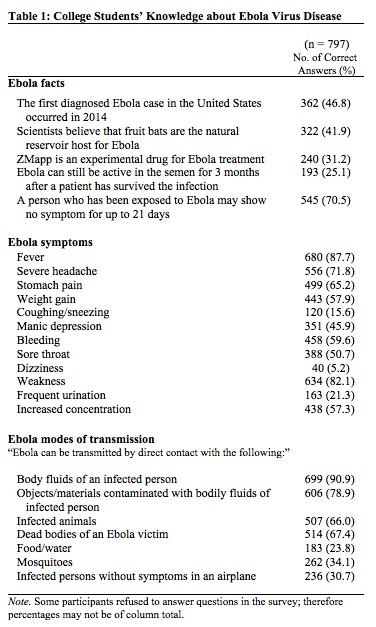
**Table 1: College Students' Knowledge about Ebola Virus Disease**

**Association Between Ebola Knowledge and News Sources Utilized**

The number of students who reported following the news about the 2014 Ebola outbreak was 438 (55%). Responses reveal the following preferred sources for Ebola information: news media (34%), social media (19%), official government websites (OGW) (11%), and family, colleague or friend (4%). Nearly one-third of participants (32%) reported utilizing a combination of these sources.

When subjects were asked to rank from least to most used information source during the 2014 Ebola outbreak, or during any national emergency such as the outbreak of a certain disease, a major industrial accident or a pandemic in the U.S., survey data revealed that OGW such as the World Health Organization (WHO), National Institutes of Health (NIH), or the Centers for Disease Control and Prevent (CDC) websites, were among the least popular sources for college students (Figure 1).

**Percentage of College Students by Main Outlet Utilized for Ebola Information (n = 664):**
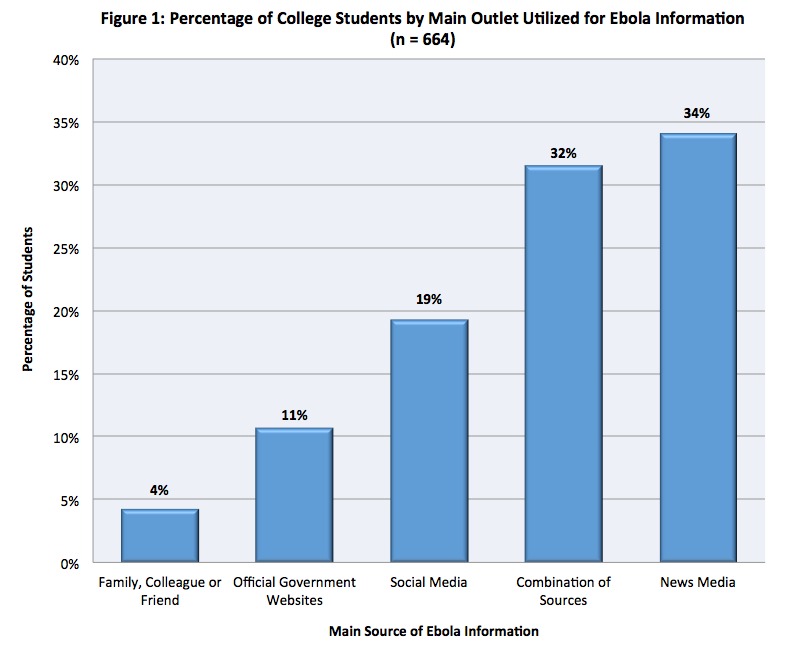

Although OGW were among the least used sources, students who reported accessing such sources had some of the highest Ebola knowledge scores. ANOVA was used to compare variances in Ebola knowledge score means across students that utilized different news sources (Figure 2).

**Comparisons of Knowledge Score Means by News Source Utilized:**
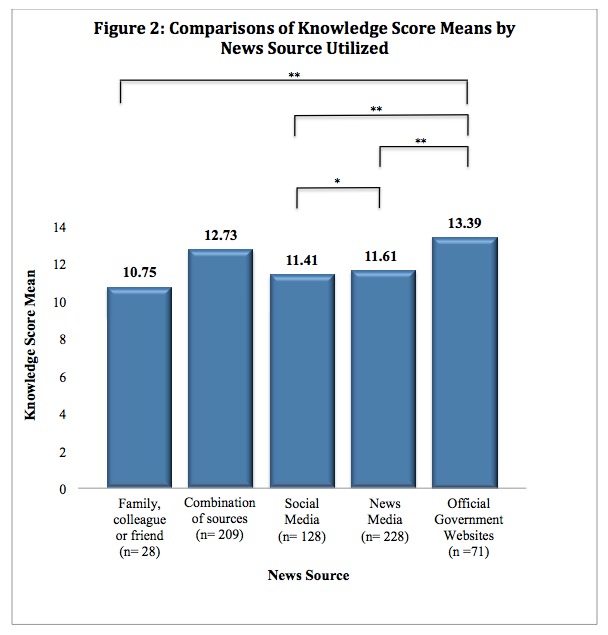
One and two asterisks indicate a p value less than 0.05 and 0.01, respectively.

The mean knowledge score for participants that utilized OGW was significantly higher than those that utilized the news media (P< .001), social media (P< .01), or family, colleagues, or friends (P< .01) as their primary EVD information source.

**Demographic Variables Associated With Ebola Knowledge**

Our study also examined differences in knowledge scores according to demographic variables such as gender, age, “biological sciences college exposure” or “non-biological sciences college exposure” academic majors, and respondents’ level of education (i.e., freshman, sophomore, junior, and senior). Respondents of ages 20 and 21, and “biological sciences college exposure” academic majors were more likely to receive higher knowledge scores than other participants, both associations were found to be statistically significant (P< .01).

For instance, 82% of students that belonged to “biological sciences college exposure” academic majors were aware that "a person who has been exposed to Ebola may show no symptoms for up to 21 days", in contrast to 67% of students that belonged to “non-biological sciences college exposure” academic majors.

Similarly, 72% (n=238) of students between ages 20 and 21, were also aware of Ebola's 21 day incubation period, in contrast to 69% among students ages 18 and 19, 70% among students ages 22 and 23, and 68% among students 24 years old and above.

Furthermore, knowledge scores according to gender were 11.7 for males (n=206), 12.4 for females (n=546), and 14.8 for transgenders (n=4). These differences in scores were not found to be statistically significant.

**Attitudes towards Ebola**

Generally, students demonstrated positive attitudes towards EVD (Mean=6.87 on a 9-point scale, SD=2.0). The highest attitude mean was observed among females (Mean=7.24, SD=1.38), followed by males (Mean=7.07, SD=1.43). Females were significantly more likely to express positive attitudes towards EVD than male participants (P= .013).

Among all respondents, 80.6% either somewhat or strongly disagreed that closing the U.S. borders was necessary to prevent an Ebola epidemic in America. Additionally, 95.8% of respondents either somewhat or strongly agreed that they would like to be assured that the Ebola vaccine is safe prior to taking it (Table 2).

**Figure:**
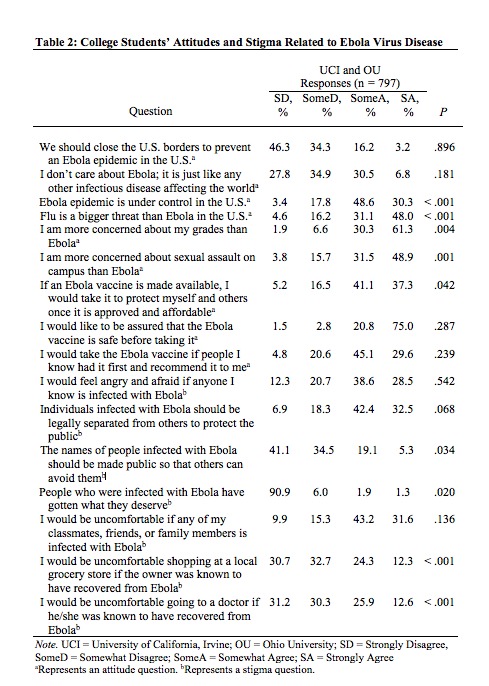
**Table 2: College Students' Attitudes and Stigma Related to Ebola Virus Disease**

**Beliefs Concerning the U.S. Government’s Involvement and Ability to Fight Ebola**

With respect to students’ beliefs concerning the U.S. Government’s involvement with the 2014 Ebola epidemic, results show that participants did not endorse the belief that Ebola is a government conspiracy created to eliminate a particular race (89%). Specifically, 87% of UCI students and 93% of OU students reported the above statement to be false. In contrast, nearly one-third of all participants were uncertain about the following statements: “There is a cure for Ebola but the government is keeping it from the public,” and “The CDC is doing all it can to control the epidemic and stop the spread of Ebola in the U.S.”

Both student populations who utilized OGW had the most positive belief (Mean=2.46, SD=0.91), whereas those that obtained Ebola information mainly from family, colleagues, or friends, presented higher distrust of the U.S. Government (Mean=1.86, SD=1.21).

**Ebola-related stigma**

Significant associations were found between demographic variables and stigmatization of persons infected with or who had recovered from EVD. Responses to survey questions such as feeling angry and afraid if those infected were known to be a familiar person (P= .01), requiring infected people’s names to be made public (P= .03), and feeling uncomfortable going to a doctor that had recovered from Ebola (P= .05), were significantly associated with respondents’ ages. This association was generally negative, with younger respondents being more likely to report higher levels of stigma towards Ebola victims.

Additionally, difference between mean stigma scores among “biological sciences college exposure” and “non-biological sciences college exposure” academic majors with regards to the statement: “Individuals infected with Ebola should be legally separated from others to protect the public,” was also found to be statistically significant with “biological sciences college exposure” academic majors expressing higher agreement with the use of legal quarantine as a method of disease control (P= .006). In comparison to male participants, females were associated with feeling more angry and afraid if those infected were known to be familiar to them (P= .001). Lastly, higher academic levels were modestly associated with lower levels of stigma with respect to consulting with a doctor known to have recovered from Ebola (P= .043).

Furthermore, multiple significant associations were found between participants’ stigma and Ebola knowledge. These associations were observed among four of the seven questions that assessed Ebola-related stigma. Results reveal a consistent pattern among students that received lower Ebola knowledge scores with presenting greater levels of stigma. This pattern was observed within the group of respondents that answered either “somewhat agree” or “strongly agree” to the following 4 statements: “People who were infected with Ebola have gotten what they deserve” (P< .001), “The names of people infected with Ebola should be made public so that others can avoid them” (P< .001), “I would be uncomfortable shopping at a local grocery store if the owner was known to have recovered from Ebola” (P< .01), and “I would be uncomfortable going to a doctor if he/she was known to have recovered from Ebola” (P< .01).

The difference in Ebola knowledge score mean of those within this group and those that presented lower stigma (i.e., “strongly disagree” or “somewhat disagree” responses to the aforementioned statements), ranged from a 1.1 to a 3.3-point difference, with the former receiving lower knowledge scores.

Among all participants, the highest stigma mean observed was for the statement regarding the use of quarantine of those infected (UCI Mean=2.96, SD=0.89 and OU Mean=3.08, SD=0.86), mean differences were not determined to be significant. Stigma means were significantly greater among UCI students for the following statements: “The names of people infected with Ebola should be made public so that others can avoid them” (UCI Mean=1.94, OU Mean=1.80; P= .03), “People who were infected with Ebola have gotten what they deserve” (UCI Mean=1.17, OU Mean=1.08; P= .02), “I would be uncomfortable shopping at a local grocery store if the owner was known to have recovered from Ebola” (UCI Mean=2.32, OU Mean=1.95; P< .001), and “I would be uncomfortable going to a doctor if he/she was known to have recovered from Ebola” (UCI Mean=2.32, OU Mean=2.0; P< .001) (Table 2).

## DISCUSSION

To our knowledge, this study is the first to assess the impact of news sources on knowledge, attitudes, beliefs, and stigma related to the 2014 EVD outbreak among college students. Findings showed great consistency in EVD knowledge between UCI and OU students. Although the vast majority reported having heard of EVD prior to taking this survey (96%), low knowledge score means acquired by both groups signified that their knowledge concerning Ebola facts, symptoms, and modes of transmission remains deficient. In addition to this gap in knowledge, misconceptions currently exist. Only a small percentage of students recognized “mosquitoes” (34%), “infected persons without symptoms in an airplane” (31%), and “food/water” (24%) as an inaccurate Ebola transmission route. These data are consistent with other reports that also reveal Ebola knowledge gap and misinformation among participants.^22^^,^ 23 ^,^ 24 ^,^ 25

Results also reveal students’ positive attitudes towards Ebola victims, their uncertainty regarding the U.S. Government’s involvement with and ability to fight the 2014 EVD outbreak, and the negative relationship between Ebola knowledge and stigma towards those infected. The results point to the key role played by the news and social media as the most prevalent sources of Ebola and other health information. These data followed similar patterns documented in the literature, which suggests that the mass media plays a crucial role in health communication during critical times.^26^^,^ 27 ^,^ 28 A noteworthy finding was the low percentage of total students that consumed official government websites (e.g. CDC, WHO, or NIH) as their main source of Ebola information (11%). Despite its low popularity among students, these sources were associated with the highest knowledge of the disease.

Previous research indicates that a distrust of health information from the CDC and other government agencies may contribute to its low popularity. As a consequence, distrust could affect audience's receptivity and responses to public health information. References have been made to health research such as the Tuskegee Study and to current government conspiracy theories related to HIV/AIDS.^37^ However, a model has been proposed for public health organizations with focus on tailoring messages to specific cultural groups prior to a pandemic.^38^ This model could serve as an essential step in emergency risk communication for it lays a foundation of trust in these sources beforehand.

In contrast, social media outlets' popularity among college students could be explained by their distinct news consumption rate patterns such as checking the news periodically, with no particular time set for news consumption. Additionally, college students news taste differs from older people's in that the former shows high levels of interest in following sports and entertainment news, whereas the latter is more interested in following news related to health and religion. Lastly, entertainment and escapism needs have been found to be positively correlated with the consumption of Internet news forms.^39^ The convenience and ease of accessibility provided by social media platforms may contribute to college students' emerging patterns of daily news consumption.

**Implications for College Students Risk Communication**

Our findings suggest that during an outbreak, most college students receive information mainly through the news and social media. However, students’ knowledge may still not be at a comprehensible level as compared to students that received information through OGW, as suggested by our results. This makes the news and social media critical outlets for delivering reliable risk communication information to this population. Although these media platforms could serve as powerful outbreak communication tools due to its instant and diverse outreach, the information spread must be monitored to ascertain the veracity of material distributed in order to dispel fear and misconceptions. College health practitioners and others in the public health sector could benefit from these findings to develop alternative strategies for reaching this target audience prior to future public health epidemics.

Alternative strategies could include OGW utilizing social media websites (e.g. Facebook, Twitter) in order to deliver occasional mass emergency alerts. This should be done by using non-technical language to describe indispensable disease facts, supplemented with educational videos in case of low literacy. Drawing on previous research, effective risk communication may decrease fear, promote self-protecting behaviors, and mitigate the spread of misinformation.^29^^,^ 30 ^,^ 31

In addition to infiltrating students' preferred sources of information during times of a health-related emergency, OGW should also be aware that the oversaturation of such messages can lead students to lessen their perceived importance of public health emergency information.^9^ For this reason, we propose the incorporation of an "emergency preparedness information tab" monitored by OGW in social media websites. This would ensure that this population, and the remaining social media users, is receiving reliable health information online. This would also prevent students from remaining misinformed while still feeling overwhelmed with passive emergency preparedness information as they engage in the web for other purposes.

**Limitations**

This study had limitations that are important to consider. First, this study was conducted at two universities and the sample of students surveyed was a fraction of the estimated 17.3 million American undergraduates.^15^ Therefore, these results may not be representative of all college campuses in America, and its interpretation from a national standpoint is limited. Second, although carefully designed, the survey was a newly developed questionnaire and additional studies are necessary to assess the validity of the measures. Lastly, this cross-sectional study assessed students’ main information sources during the Ebola outbreak. Therefore, we cannot infer associations found to students’ daily health information sources.

## PUBLIC HEALTH IMPLICATIONS

Our study demonstrated that reliable sources for EVD information such as OGW could significantly increase college students’ knowledge of Ebola. However, OGW is among the least popular source of outbreak information amid this population. Therefore, public health authorities should revamp risk communication strategies to infiltrate into popular sources among students (e.g. news and social media) ensuring the dissemination of reliable information to this target audience during public health emergencies.

In light of the ongoing Zika virus epidemic, an emerging arbovirus that has been associated with microcephaly in children born to infected mothers and Guillain-Barré syndrome in infected adults,^32^ recent nationwide poll of 1,004 adults conducted between March 17 and 26 of 2016, revealed that only 43% of Americans age 18-29 have heard about the Zika virus.^33^ This study also reveals large gaps in respondents' knowledge of its modes of transmission, with only 57% of those aware of the virus knowing that it can spread through sexual intercourse with an infected person. In comparison to our study results, although a greater level of awareness for EVD exists among college students (96%), similar gaps in knowledge of EVD modes of transmission was also identified, with only 34% of students knowing that EVD cannot spread through mosquitoes. As many as 200,000 Americans are expected to travel to Rio de Janeiro for the Summer Olympics 2016,^34^ therefore the possibility of Americans contracting the virus while in Brazil, Zika's biggest hotspot, cannot be underestimated.

The Ebola outbreak and the current Zika epidemic should serve as a reminder that the more interconnected our globalized world grows, the more vulnerable we become to pathogens once thought to be locally confined to certain areas of the globe. Whether the next public health threat will be a super bacteria, a novel mosquito-borne disease, or an airborne disease, it remains unsure. However, we can say with certainty that communication strategies to college students should be improved before disaster strikes.

## COMPETING INTERESTS

The authors have declared that no competing interests exist.

## CORRESPONDENCE

Miryha G. Runnerstrom

E-mail: miryha@uci.edu
